# White coat and masked effects depend on blood pressure level and time of blood pressure measurement

**DOI:** 10.3389/fmed.2025.1550418

**Published:** 2025-05-13

**Authors:** Matthieu Halfon, Patrick Taffe, Gregoire Wuerzner

**Affiliations:** ^1^Service of Nephrology and Hypertension, Lausanne University Hospital and University of Lausanne, Lausanne, Switzerland; ^2^Transplantation Center, Lausanne University Hospital, Lausanne, Switzerland; ^3^Division of Biostatistics, Center for Primary Care and Public Health (Unisanté), University of Lausanne, Lausanne, Switzerland

**Keywords:** white coat effect, agreement, limits of agreement, differential bias, proportional bias, blood pressure, circadian rhythm, hypertension

## Abstract

**Background:**

A total of 24 h ambulatory blood pressure monitoring (ABPM) offers enhanced accuracy for evaluating true blood pressure and associated risks compared to office blood pressure (OBP). However, conflicting results have been reported in studies comparing the two settings, largely due to the statistical bias introduced by the mean difference calculation using the Bland and Altman method, especially if the inherent circadian variation of blood pressure is not considered. This study aimed to assess the difference between OBP and ABPM using a refined statistical approach while accounting for circadian variations at different blood pressure levels.

**Methods:**

Multilevel/mixed-effects harmonic regression models were employed to estimate mean 24 h systolic and diastolic blood pressure profiles. The bias plot method, with ABPM as the reference, was used to calculate the OBP- daytime ABPM difference.

**Results:**

A total of 647 participants were included with a median of 63 measurements per individual, with most OBP measurements conducted between 8 a.m. and 10 a.m. Analysis showed individual average systolic OBP-ABPM differences ranging from +10 to −30 mmHg and diastolic differences ranging from +20 to −60 mmHg. As ABPM values increase, the patients tend to exhibit a masked effect. Normotensive individuals on ABPM exhibited a white coat effect phenotype, with systolic OBP-ABPM differences ranging from +6 to +9 mmHg. Conversely, hypertensive patients displayed a modest white coat effect for those at the lower hypertension limit and a pronounced masked effect for those at higher hypertension levels. The reduced circadian blood pressure variation observed in hypertensive patients, characterized by a nadir shift to later in the day, contributed to this divergence.

**Conclusion:**

Differences between OBP and ABPM depend on mean ABPM blood pressure levels. OBP tends to overestimate in normotensive and underestimate in hypertensive patients. Differences in circadian variation between these groups contribute to the variance.

## Introduction

Office blood pressure (OBP) measurement is one of the most frequently performed medical procedure during outpatient visits. An accurate measurement is crucial for diagnosing and treating hypertension, a highly prevalent condition in the population and a significant risk factor for cardiovascular diseases (1). However, it is widely recognized that blood pressure (BP) measurements obtained at hospitals or medical offices may not always provide reliable diagnostic results. In fact, BP readings can vary significantly depending on whether they are taken in a clinical setting, at home, or through 24 h ambulatory blood pressure monitoring (ABPM) (2, 3). Currently, there is ongoing debate among experts regarding the optimal approach to blood pressure monitoring. As some experts advocate for ABPM to be utilized as the primary method (4, 5).

A recent meta-analysis has confirmed that attended measurements (taken by medical staff) generally yielded higher BP values compared to unattended measurements (taken by patients themselves) (2). This phenomenon is commonly referred to as the “white coat effect” (WCE). The WCE is often responsible for the higher OBP readings observed in a majority of patients when compared to average 24 h ABPM measurements (6). WCE should not be confused with white coat hypertension, the latter describing a patient who is hypertensive on OBP but normotensive with ABPM.

However, it is important to note that for some patients, the opposite phenomenon can occur, known as the “masked effect” (MASE) (7). In this case, OBP readings are lower than ABPM. Both WCE and MASE are now widely recognized BP phenotypes and taken into consideration in clinical practice. Furthermore, the extent of WCE or MASE holds significance, as indicated by studies revealing that patients with a high WCE (> 30 mmHg) face a 2-fold higher risk of mortality compared to their counterparts (8).

Therefore, considering the WCE and MASE when evaluating BP readings is useful for a comprehensive understanding of a patient’s cardiovascular health and overall prognosis and to adequately assesses the difference between OBP and ABPM.

Many studies have tried to quantify the WCE and MASE based on the Bland and Altman method (9–11). However, recently the Bland and Altman method has been shown to rely on strong statistical assumptions which unfortunately are often violated in practice and therefore is likely to provide biased results (9, 12). Consequently from a methodological point of view these effects have not been adequately quantified (9, 12). In addition, BP follows a circadian pattern with an important nadir during the night (13). Consequently, it is important to exclude ABPM measurements taken during the night when assessing WCE and MASE. Finally, the difference between OBP and ABPM (and consequently the WCE and MASE) might depend on the patient’s true average BP level, some patients exhibiting systematically lower than population average BP values and others higher than population average.

The goal of this study was to use a new statistical method to quantify the WCE and MASE that overcome the main limitations of the Bland and Altman method (12).

In addition, taking into consideration the circadian profile of longitudinal BP values we used a hierarchical/mixed-effects harmonic regression models to improve stratification of the participant into BP levels based on their observed systolic BP (SBP) and diastolic BP (DBP) values.

## Materials and methods

### Study design

#### Ethics

The ethical commission of canton Vaud^1^ was consulted before the trial was started. As this study is a secondary analysis of an already anonymous database collected for clinical purpose, no formal informed consent was necessary in accordance with Swiss Human Research *ACT*.

#### Devices

To avoid the biases inherent to the comparison of two sets of measurement taken by different measurement methods (automatic devices vs manual cuffs), all BP measurements were taken by the same device using the oscillometric method. All measurements were done using the validated DIASYS 3 (Novacor, France) ABPM monitoring device. The device was used according to the manufacturer recommendations and the European practice guidelines for ABPM monitoring (4).

#### Participants

All patients who underwent an ABPM in our tertiary hypertension center from 1 January 2019 to 31 December 2020 were included retrospectively and in a chronological order. Exclusion criteria were less than 21 daytime BP measurements by the device and age younger than 18.

#### Measurements

The first two BP measurements were taken by the nurse trained in BP measurement, following the current recommendation of the European Society of hypertension (ESH),with the DIASYS 3 connected to a mercury-free validated sphygmomanometer (A&D, UM-101) with a Y-tube using the oscillometric method (4). In brief :

• Measurements were taken in a quiet room at a comfortable temperature.
• Patients refrained from smoking, caffeine intake, food, and physical activity for at least 30 min prior to the measurement.
• Patients remained seated and relaxed for 3–5 min before the measurement.
• No talking was allowed by either the patient or staff during or between measurements.

Posture during Measurement:

• The patient was seated with their back supported by a chair.
• Legs were uncrossed with feet flat on the floor.
• The arm was bare, resting on a table, with the mid-arm positioned at heart level.

**FIGURE 1:**
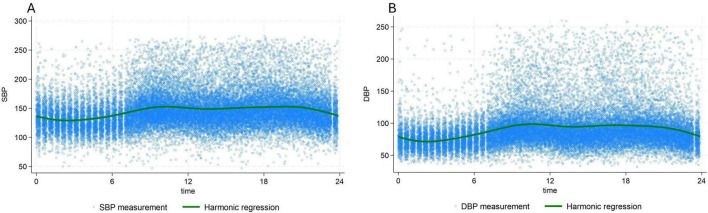
24 h, circadian variation of systolic blood pressure **(A)** and diastolic blood pressure **(B)** Green line: population averaged curve based on the multilevel/mixed harmonic regression model. DBP, diastolic blood pressure; SBP, systolic blood pressure.

Other subsequent measurements were done automatically every 20 min by the DIASYS 3, for 24 h. All the measurements except the two first were done in an ambulatory setting. Therefore, we conjectured that the first two BP measurements were representative of OBP measurements and subsequent measurements of ABPM, since the possible stress due to the presence of the nurse should have disappeared by the third measurement (14). Because of the circadian variation of BP, with lower BP during nighttime (Figure 1), only measurements taken between 7 a.m. and 10 p.m. were used for the analyses to compare OBP and ABPM difference.

#### BP categories

We defined subgroups of normotensive and hypertensive patients based on the standard definitions (4):

based on 24 h ABPM:

• Normotensive patients were defined as a systolic ABPM < 130 mmHg and diastolic ABPM < 80 mmHg.
• Hypertensive patients were defined as a systolic ABPM ≥ 130 mmHg or diastolic ABPM ≥ 80 mmHg.

based on OBP:

• Normotensive patients were defined as systolic OBP < 140 mmHg and diastolic OBP < 90 mmHg.
• Hypertensive patients were defined as systolic OBP ≥ 140 mmHg or diastolic OBP ≥ 90 mmHg.
• Dipping was defined as a decrease of nighttime systolic BP (SBP) of more than 10% of daytime SBP.

In addition, we also considered subgroups of BP level defined by the six following categories.

Given the high variability of individual repeated measurements, to get better estimates of the “true average BP” we used a sophisticated statistical modeling technic (see statistical method) to estimate of the true average individual mean SBP and DBP values using the BP value of the 24 h period. The “true average”24 h BP value was used stratify in patients in the six sub-groups (Supplementary Figure 1).

• Group 1: 100 ≤ true average 24 h SBP < 120 mmHg or 60 ≤ true average 24 h DBP < 80 mmHg
• Group 2: 120 ≤ true 24 h average SBP < 130 mmHg or 80 ≤ true 24 h average 24 h DBP < 85 mmHg
• Group 3: 130 ≤ true average 24 h SBP < 140 mmHg or 85 ≤ true average 24 h DBP < 90 mmHg
• Group 4: 140 ≤ true average 24 h SBP < 160 mmHg or 90 ≤ true average 24 h DBP < 100 mmHg
• Group 5: 160 ≤ true average24 h SBP < 180 mmHg or 100 ≤ true average 24 h DBP < 110 mmHg
• Group 6: true average SBP ≥ 180 mmHg or true average DBP ≥ 110 mmHg

(Note that there was no patient in the category: average SBP < 100 or 60 < average DBP), and as groups have been assessed sequentially each individual belongs to a single group, i.e., the last group meeting his criteria).

#### Statistical analyses

##### Method for stratification by BP level

The SBP and DBP population averaged 24 h profiles (Figure 1), were estimated using multilevel/mixed effects harmonic regression models (15, 16). These models are very flexible and allow the capture of the circadian pattern of BP values using a series of harmonics (sine and cosine terms in a Fourier expansion). Thanks to the use of random effects, even the individual profiles are available (Supplementary Figure 1). The great advantage of this methodology is that it allows accounting for both the circadian pattern of BP and, importantly, measurement errors in the analyses. Therefore, it produces individual mean SBP and DBP values which are more robust than the simple individual 24 h means. Finally, based on these “modeled” means, individuals have been classified into the BP level defined above.

##### Method for OBP and ABPM comparison

To assess the WCE and MASE a recently published statistical technic has been used, which overcomes the important limitations of the Bland and Altman method (9, 10, 12). SBP and DBP were analyzed separately. Very briefly, like for population averaged 24 h profiles above, this new modeling technic uses the individual repeated measurements to estimate the true latent mean BP value for each individual (called BLUP, for best linear unbiased prediction). It is important to emphasize that these true latent individual mean BP values cannot be computed by simply calculating the average mean value in each individual because of measurement errors; rather it has to rely on a modeling technic.

Only daytime BP values have been used in the comparison of OBP with ABPM to remove the circadian pattern and simplify analyses. A figure, called “Bias plot,” representing the data in a scatter plot is drawn. The Bland and Altman limits of agreement was also used to compute the OBP-ABPM difference.

## Results

### Study population and BP measurements

A total of 647 participants were included in our study with a median number of 63 over 24 h and 42 daytime measurements per individual. Of the 647 ABPMs performed, 216 (33%) were conducted for the diagnosis of hypertension, 358 (55%) for therapy monitoring, and 46 (7%) for other reasons. In 27 cases (4%), the indication for ABPM was unknown.

The median age of patients was 56 years, (IQR:45–66) and 372 (58%) were male. A total of 462 patients (71%) were classified as hypertensive based OBP, while 569 patients (88%) were identified as hypertensive based on ABPM. Among them, 38 patients (6%) were considered hypertensive according to OBP, but normotensive based on ABPM, whereas 89 patients (13%) were classified as normotensive by OBP but hypertensive according to ABPM (Table 1). There was no sex difference between hypertensive and normotensive patients (*P* = 0.5). Only 48% of patients had a preserved dipping pattern. Most OBP were recorded between 8 a.m. to 10 a.m. (Supplementary Figure 2), which corresponds to the time slot when ABPM devices were put on patients.

**TABLE 1:** Characteristics of the patients.

Variable	All patients (*N* = 647)	Women (*N* = 273)	Men (*N* = 72)
Median age (years)^a^ (IQR1–IQR3)	56 (45–66)	57 (45–67)	55 (45–64)
Sex male/female (%)^b^	372 (58%)/273 (42%)	–	–
Median ABPM systolic blood pressure (mm Hg) (IQR1–IQR3)	141 (125–160)	141 (126–160)	141 (126–159)
Median daytime ABPM SBP (mm Hg) (IQR1–IQR3)	145 (130–165)	146 (129–166)	145 (131–164)
Median nighttime ABPM SBP (mm Hg) (IQR1–IQR3)	132 (119–148)	133 (119–149)	131 (118–147)
Median ABPM diastolic blood pressure (mm Hg)	84 (72–99)	84 (71–99)	84 (72–98)
Median daytime ABPM DBP (mm Hg)	89 (77–104)	89 (76–106)	89 (77–103)
Median nighttime ABPM DBP (mm Hg)	76 (86–87)	76 (66–87)	76 (66–87)
Median OBP systolic blood pressure (mm Hg) (IQR1–IQR3)	148 (136–162)	148 (135–163)	148 (137–161)
Median OBP diastolic blood pressure (mm Hg) (IQR1–IQR3)	88 (80–97)	87 (78–95)	89 (80–97)
Hypertensive patient by 24 h-ABPM, *n* (%)	569 (88%)	236 (86%)	328 (82%)
Hypertensive patients by OBP, *n* (%)	462 (71%)	211 (77%)	302 (81%)
Mean BMI (IQR1–IQR3)	27 (24–30)	27 (24–30)	27 (23–31)

^a^Missing data for 35 patients.

^b^Missing data for sex for two patients. ABPM, ambulatory blood pressure; BMI, body mass index; IQR, interquartile range; OBP, office blood pressure.

**FIGURE 2:**
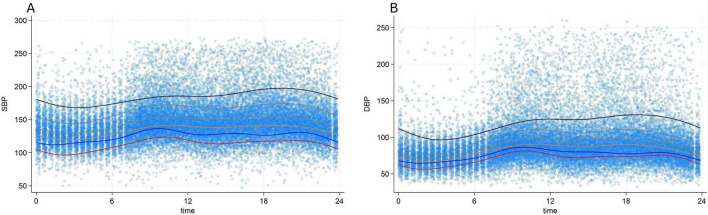
24 h, circadian variation of systolic blood pressure **(A)** and diastolic blood pressure **(B)** according to blood pressure categories. Red line: 100 ≤ true average 24 h SBP < 120 mmHg or 60 ≤ true average 24 h DBP < 80 mmHg, Blue line: 120 ≤ true average 24 h SBP < 130 mmHg or 80 ≤ true average 24 h DBP < 85 mmHg, Orange line: 130 ≤ true average 24 h SBP < 140 mmHg or 85 ≤ true average 24 h DBP < 90 mmHg, Brown line: 140 ≤ true average 24 h SBP < 160 mmHg or 90 ≤ true average 24 h DBP < 100 mmHg, Grey line: 160 ≤ true average 24 h SBP < 180 mmHg or 100 ≤ true average 24 h DBP < 110 mmHg, Black line: true average 24 h SBP ≥ 180 mmHg or true average 24 h DBP ≥ 110 mmHg, DBP, diastolic blood pressure; SBP, systolic blood pressure.

The median systolic and diastolic OBP were 148 mmHg, (IQR:136–162) mmHg and 88 mmHg, (IQR:90–97) mmHg, respectively. For ABPM median daytime SBP was 145 mmHg, (IQR:130–165) and median daytime DBP 89 mmHg, (IQR:77–104) (Table 1). Analysis of profiles of BP values over time showed a clear circadian pattern with lower BP values during the night (Figure 1). The circadian pattern could also be observed in the different subgroups of BP phenotypes with possibly an attenuated difference between day and night values in patients with an elevated average BP level (Figure 2).

### Difference between OBP and ABPM (all patients)

**FIGURE 3:**
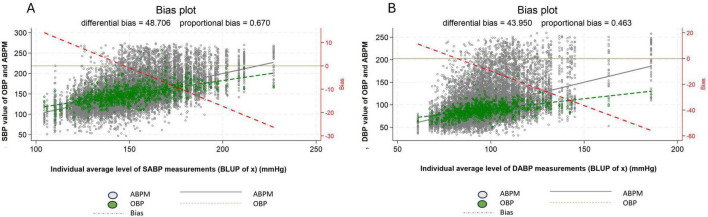
Difference in measured blood pressure between office blood pressure and daytime ambulatory blood pressure, systolic blood pressure **(A)** diastolic blood pressure **(B)**. For systolic blood pressure mean OPB and mean daytime ABPM difference: the differential bias was 48.0 mmHg and the proportional bias: 0.7 mmHg. For diastolic blood pressure mean OPB and mean daytime ABPM difference: the differential bias was 44 mmHg and the proportional bias: 0.5 mmHg. The left y-axis represents the ABPM (in black) and OBP (in green) measurements and the x-axis the BLUP (i.e., the best possible estimation of the individual mean value). In addition, two regression lines have been computed and added to the scatter plot, representing the relation between ABPM (in black) and between OBP (in green) and the BLUP. The vertical difference between the two regression lines, referred to as “bias,” represents the discrepancy between the two measurement devices (i.e., ABPM and OBP), and allows one to assess the WCE/MASE. a third regression line (red dash-dotted) representing the bias (i.e., the difference between the ABPM and OBP regression lines) has been added to the plot. The distance between the ABPM and OBP regression lines can be easily read on the right y-axis for each value of the BLUP. For example, from the bias plot a patient with an average daytime systolic ABPM of 105 mmHg had a white coat effect (WCE) of about 10 mmHg and a patient with an average daytime systolic ABPM of 200 mmHg a masked effect (MASE) of about - 20 mmHg. Regarding diastolic blood pressure, a patient with an average daytime diastolic ABPM of 60 mmHg had a WCE of about 10 mmHg and with an average diastolic ABP of 115 mmHg a MASE of about - 10 mmHg. White dot: individual blood pressure per subject (ABPM), Green dot: individual blood pressure per subject (OBP), Dash red line: regression line of average difference between OBP and ABPM values, Plain black line: mean ABPM value (all patients), Dash green line: mean OBP value (all patients), ABPM, daytime ambulatory blood pressure; OBP, office blood pressure; SBP, systolic blood pressure; DBP, diastolic blood pressure.

Comparison of the two regression lines for OBP and ABPM on the bias plot shows that the difference (or bias) between individual average systolic OBP and individual average daytime systolic ABPM ranged from +10 to −30 mmHg, and between +20 and −60 mmHg for individual average diastolic OBP and individual average daytime diastolic ABPM (Figures 3A, B).

Patients with a true latent systolic ABPM below 150 mmHg (referred to as BLUP of x on the bias plot figure) displayed an increasing WCE (OBP > ABPM), and patients with a true latent systolic ABPM higher than 150 mmHg displayed an increasing MASE (OBP < ABPM). A similar pattern can be observed for DBP.

### Difference between OBP and ABPM in normotensive vs hypertensive patients

**FIGURE 4:**
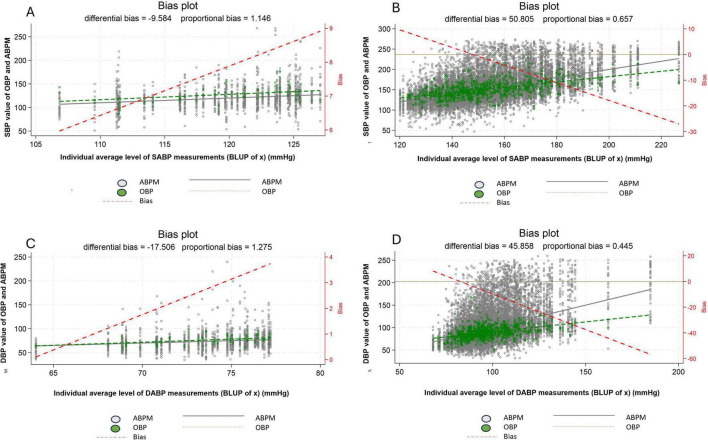
Difference between office blood pressure and daytime ambulatory blood pressure, between normotensive **(A,C)** and hypertensive patients **(B,D)** for systolic blood pressure **(A,B)** and diastolic blood pressure **(C,D)**. White dot: individual blood pressure per subject (ABPM), Green dot: individual blood pressure per subject (OBP), Dash red line: regression line of average difference between OBP and ABPM values, Plain black line: mean ABPM value (all patients), Dash green line: mean OBPM value (all patients), ABPM, daytime ambulatory blood pressure measurement; OBP, office blood pressure; SBP, systolic blood pressure; DBP, diastolic blood pressure.

Considering normotensive patients (based of 24 h ABPM), the difference between individual average systolic OBP and individual average systolic ABPM ranges from +6 to +9 mmHg and therefore illustrates a purely WCE (Figure 4A). Whereas in hypertensive patients the difference between individual average systolic OBP and individual average systolic ABPM ranges from +10 to about −30 mmHg. Thereby showing a modest WCE for individuals at the lower limit of the definition of hypertensive patients and a clear MASE for individuals in the upper range of hypertension (Figure 4B). A similar pattern can be observed for diastolic blood pressure (Figures 4C, D).

### Difference between OBP and ABPM by groups of BP level

**FIGURE 5:**
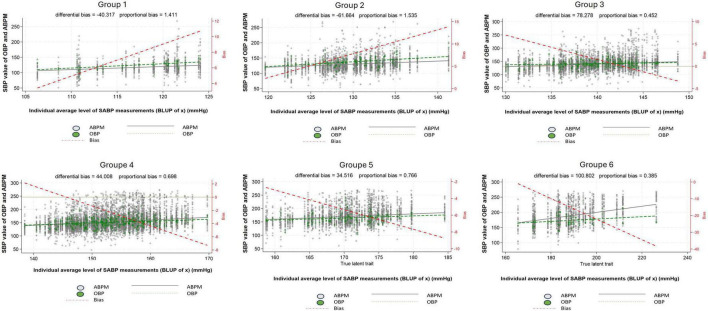
Difference between systolic office blood pressure and systolic daytime ambulatory blood pressure according to the 24 h blood pressure level. Group 1: 100 ≤ true average SBP < 120 mmHg or 60 ≤ true average DBP < 80 mmHg, Group 2: 120 ≤ true average SBP < 130 mmHg or 80 ≤ true average DBP < 85 mmHg, Group 3: 130 ≤ true average SBP < 140 mmHg or 85 ≤ true average DBP < 90 mmHg, Group 4: 140 ≤ true average SBP < 160 mmHg or 90 ≤ true average DBP < 100 mmHg, Group 5: 160 ≤ true average SBP < 180 mmHg or 100 ≤ true average DBP < 110 mmHg, Group 6: true average SBP ≥ 180 mmHg or true average DBP ≥ 110 mmHg, White dot: individual blood pressure per subject (ABPM), Green dot: individual blood pressure per subject (OBP), Dash red line: regression line of average difference between OBP and ABPM values, Plain black line: mean ABPM value (all patients), Dash green line: mean OBP value (all patients), ABPM, daytime ambulatory blood pressure; OBP, office blood pressure; SBP, systolic blood pressure.

Now, considering the six groups of BP level (defined based on our multilevel/mixed harmonic regression modeling) and focusing only on SBP, the WCE is apparent in patients belonging to the first two groups (Group 1 and 2) and the MASE in patients belonging to the last two groups (Group 5 and 6) (Figure 5). A similar pattern can be observed for DBP (Supplementary Figure 3).

### Difference between OBP and ABPM using Bland and Altman method

When OBP and ABPM difference was computed using the Bland and Altman method, the results shown a purely MASE for all BP groups level (Supplementary Figure 4).

The mean OBP-ABPM differences were 0 mmHg (95% CI: −40 to +40) and −6.3 mmHg (95% CI: −59 to +50) for group 1 and 2, respectively and −25.0 mmHg (95% CI: −75 to +50) and −13.5 mmHg (95% CI: −59 to +45) for group 5 and 6, respectively.

## Discussion

Our findings, based on our complex statistical modeling technics reveal that the difference between OBP and daytime ABPM measurements depends essentially on BP level. This difference is positive for subgroups of individuals exhibiting a BP level with low BP values, thereby demonstrating WCE, whereas it is negative for subgroups of individuals exhibiting high values of BP level, thereby demonstrating MASE. Moreover, using only the Bland and Altman method to assess WCE and MASE failed to capture their variation in relation to individual BP values.

Our study demonstrates that normotensive patients commonly exhibit a WCE, whereas hypertensive patients tend to display a MASE. One possible explanation for the higher prevalence of the WCE in normotensive patients is that they often undergo ABPM as part of the diagnostic process for hypertension. As a result, these patients may be more susceptible to the fear or anxiety associated with being diagnosed with hypertension. In contrast, patients with already elevated BP levels may not experience the same level of concern during OBP measurements (17). Additionally, some ABPM tests performed in normotensive patients may have been prompted by suspicion of the white coat hypertension (17). In both scenarios, these “normotensive” patients may exhibit increased sympathetic drive, leading to an elevation in BP measurements taken in the office compared to ambulatory BP readings (8).

It is noteworthy that the circadian pattern of BP undergoes changes based on the true average BP value, wherein patients with higher BP values tend to have reduced circadian variations and more salt sensitivity (19). Therefore, the fluctuation of BP throughout the day should also be considered when assessing OBP. In our study, the majority of baseline OBP measurements were conducted between 8 a.m. and 10 a.m. when BP is at its peak in normotensive patients (Figure 2). However, in hypertensive patients, the peak of BP is delayed, and BP tends to rise consistently throughout the daytime (Figure 2). As a result, baseline OBP measurements taken between 8 a.m. and 10 a.m. may appear lower compared to BP measurements taken at other times of the day in this group of patients. This correlation between BP value and circadian pattern variations could help elucidate the underlying mechanisms behind the differential effects observed in normotensive and hypertensive patients.

We would like to highlight that in our study each individual was classified into a BP level group based on his predicted average latent BP value determined by the harmonic regression model (rather than based simply on his observed average ABPM values), which allows one to take into account the circadian variation of BP in each patient. Considering that BP naturally fluctuates in a circadian rhythm, it is not surprising that measurements obtained through a 24 h monitoring device would also be influenced by these variations. However, it is crucial to recognize that the circadian variation of blood pressure is not uniform and depends on the mean BP value (Figure 2).

Often in studies, the OBP-ABPM difference is typically reported by using the Bland and Altman method, presented as a mean OBP-ABPM difference (11, 20–22) However, it is important to highlight that OBP-ABPM difference is generally not uniform and its amplitude varies along the range of BP values considered. Therefore, a more accurate and informative approach would be to consider the variability of OBP-ABPM difference within a specific range of BP values.

Office blood pressure-ambulatory blood pressure monitoring difference within the analyzed range could help provide a more comprehensive understanding of WCE and MASE phenomena. This is illustrated in our study by comparing the results between the Bias plot and the Bland and Altman method. Indeed, using the Bias plot, we were able to demonstrate a smooth transition from WCE for patients with lower BP level values to MASE for patients with higher BP level values. However, the results obtained using the Bland and Altman method only reflect MASE for all BP level groups and do not capture the WCE for groups with lower BP values. Unfortunately, this finding considerably undermines the validity of the results from studies that rely solely on the Bland and Altman method, especially concerning BP values distant from the mean BP (25, 26).

## Conclusion

With the advancements in cuffless devices enabling intermittent or continuous BP measurements around the clock and for several days, the future of BP monitoring is undergoing a significant transformation. A paradigm shift is imminent, where patients will easily assess their BP at any hours. Consequently, considering the time of day for BP measurements in alignment with the circadian variation of BP will be crucial for accurate analysis of results (27). Utilizing our method, we were not only able to assess the circadian variation of BP through daytime and nighttime measurements but also take into account the patient’s individual hour-to-hour circadian variation of BP. By doing ABPM throughout the entire daytime, we can gather comprehensive data that captures the unique fluctuations in BP specific to each patient. Furthermore, the rapid advancements in the field of smartwatches or connected devices hold the potential to enable precise assessment of BP at a granular level in the near future. This approach holds great promise in advancing the field toward more precise and tailored medical interventions. Additionally, circadian differences in BP patterns, such as the abolition of the dipping pattern, have been observed in patients and have already been associated with cardiovascular risk (28). Further studies are needed to investigate the correlation between circadian variation and cardiovascular risk.

## Strength and limitation

A major strength of our study lies in the use of a new and more appropriate statistical methodology than previous studies based on The Bland and Altman method. Firstly, our study provided more accurate and precise data on the difference between OBP and 24 h ABPM compared to previous studies. This enhanced accuracy allows for a more comprehensive understanding of the OBP-ABPM difference. Secondly, we were able to account for the influence of the circadian rhythm on BP measurements at a patient level to determine “true” BP level, which adds depth and context to our findings. However, a limitation in our study is the absence of patients’ self-reported activity diaries. Consequently, a fixed timeframe was employed to define daytime and nighttime. It’s important to acknowledge that we cannot exclude the possibility that some patients may have slept during the daytime or been awake during the nighttime. Another limitation is the absence of data regarding the use of anti-hypertensive medications and other possible confounding factors sur as kidney function or diabetes or salt intake. Future studies incorporating medication data and variable affection the circadian rhythm would be beneficial in exploring the relationship between anti-hypertensive drugs and circadian BP variations. Finally, a third limitation is the use of only two office blood pressure (OBP) measurements, rather than the three recommended by the ESH guidelines.

## Data Availability

The anonymized data are available upon reasonable request for a collaborative research project. Please contact the corresponding author for all requests regarding data sharing.

## Footnotes

1
www.cer-vd.ch

## Supplementary material

Supplementary Figure 1 Illustration of 24 h, circadian variation of systolic blood pressure **(A)** and diastolic blood pressure **(B)** for three randomly selected patients. The data for three randomly selected patients have been graphed in a scatter plot depicting the circadian relationship of systolic blood pressure (left panel) and diastolic blood pressure (right panel) with time. In addition, dashed lines representing the average “true estimated” 24 h BP individual values have been superimposed. The “true estimated” 24 h BP value have been computed using a harmonic regression model. Note that the value of the “true estimated” 24 h BP is different than the mean 24 hs BP value. These individual average “true estimated” values define natural BP level. Green lines represent the average “true estimated” blood pressure values of the overall 647 patients include.

Supplementary Figure 2 Distribution of time of the first office blood pressure measurement.

Supplementary Figure 3 Difference between diastolic office blood pressure and diastolic daytime ambulatory blood pressure by groups of BP level. Group 1: 100 ≤ true average SBP < 120 mmHg or 60 ≤ true average DBP < 80 mmHg, Group 2: 120 ≤ true average SBP < 130 mmHg or 80 ≤ true average DBP < 85 mmHg, Group 3: 130 ≤ true average SBP < 140 mmHg or 85 ≤ true average DBP < 90 mmHg, Group 4: 140 ≤ true average SBP < 160 mmHg or 90 ≤ true average DBP < 100 mmHg, Group 5: 160 ≤ true average SBP < 180 mmHg or 100 ≤ true average DBP < 110 mmHg, Group 6: true average SBP ≥ 180 mmHg or true average DBP ≥ 110 mmHg, White dot: individual blood pressure per subject (ABP), Green dot: individual blood pressure per subject (OBP), Dash red line: regression line of average difference between OBP and ABPM values, Plain black line: mean ABPM value (all patients), Dash green line: mean OBP value (all patients), ABPM, daytime ambulatory blood pressure; OBP, office blood pressure; DBP, diastolic blood pressure. For diastolic blood pressure only blood pressure value ranging from 40 to 150 mmHg were considered.

Supplementary Figure 4 Difference between systolic office blood pressure and systolic daytime ambulatory blood pressure by groups of BP level using the Bland and Altman methodology. Group 1: 100 ≤ true average SBP < 120 mmHg or 60 ≤ true average DBP < 80 mmHg, Group 2: 120 ≤ true average SBP < 130 mmHg or 80 ≤ true average DBP < 85 mmHg, Group 3: 130 ≤ true average SBP < 140 mmHg or 85 ≤ true average DBP < 90 mmHg, Group 4: 140 ≤ true average SBP < 160 mmHg or 90 ≤ true average DBP < 100 mmHg, Group 5: 160 ≤ true average SBP < 180 mmHg or 100 ≤ true average DBP < 110 mmHg, Group 6: true average SBP ≥ 180 mmHg or true average DBP ≥ 110 mmHg, Violet line: mean difference between OBP and ABPM, Red lines: 95% limits of agreements, Green line: regression line. OBP, office blood pressure; ABPM, daytime ambulatory blood pressure.
