# Supplementary figures and images for: White coat and masked effects depend on blood pressure level and time of blood pressure measurement
Source: Front Med (Lausanne). 2025 May 13;12:1550418. doi: 10.3389/fmed.2025.1550418 (PMC12106515; doi:10.3389/fmed.2025.1550418)

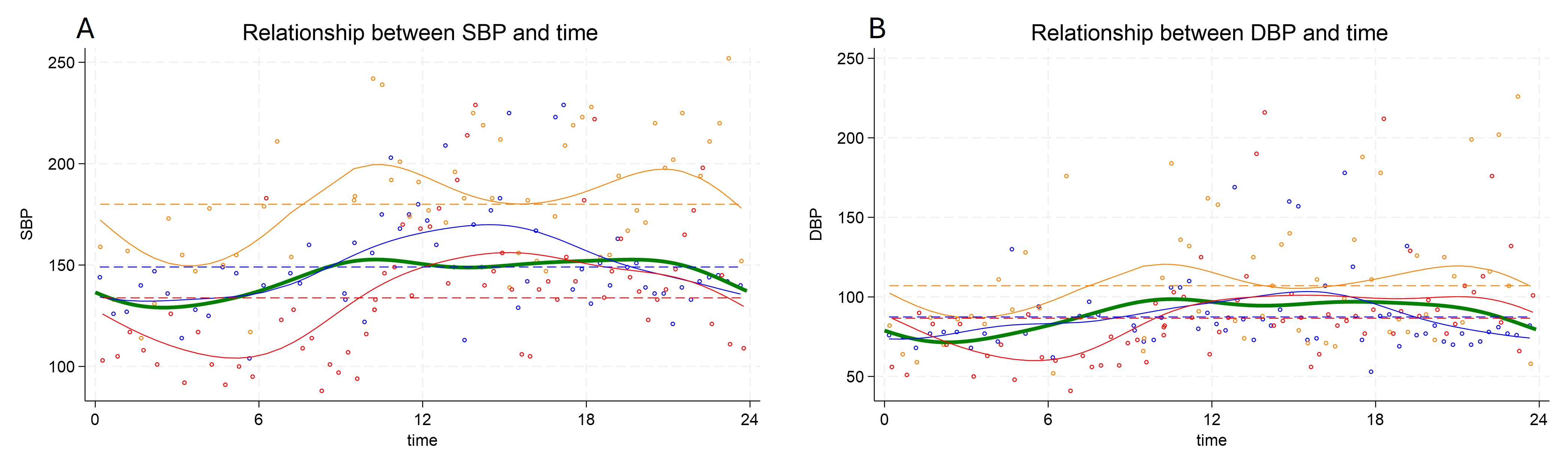

Supplement: Supplementary Figure 1 — Illustration of 24 h, circadian variation of systolic blood pressure (A) and diastolic blood pressure (B) for three randomly selected patients. The data for three randomly selected patients have been graphed in a scatter plot depicting the circadian relationship of systolic blood pressure (left panel) and diastolic blood pressure (right panel) with time. In addition, dashed lines representing the average “true estimated” 24 h BP individual values have been superimposed. The “true estimated” 24 h BP value have been computed using a harmonic regression model. Note that the value of the “true estimated” 24 h BP is different than the mean 24 hs BP value. These individual average “true estimated” values define natural BP level. Green lines represent the average “true estimated” blood pressure values of the overall 647 patients include. [file Image_1.jpeg]

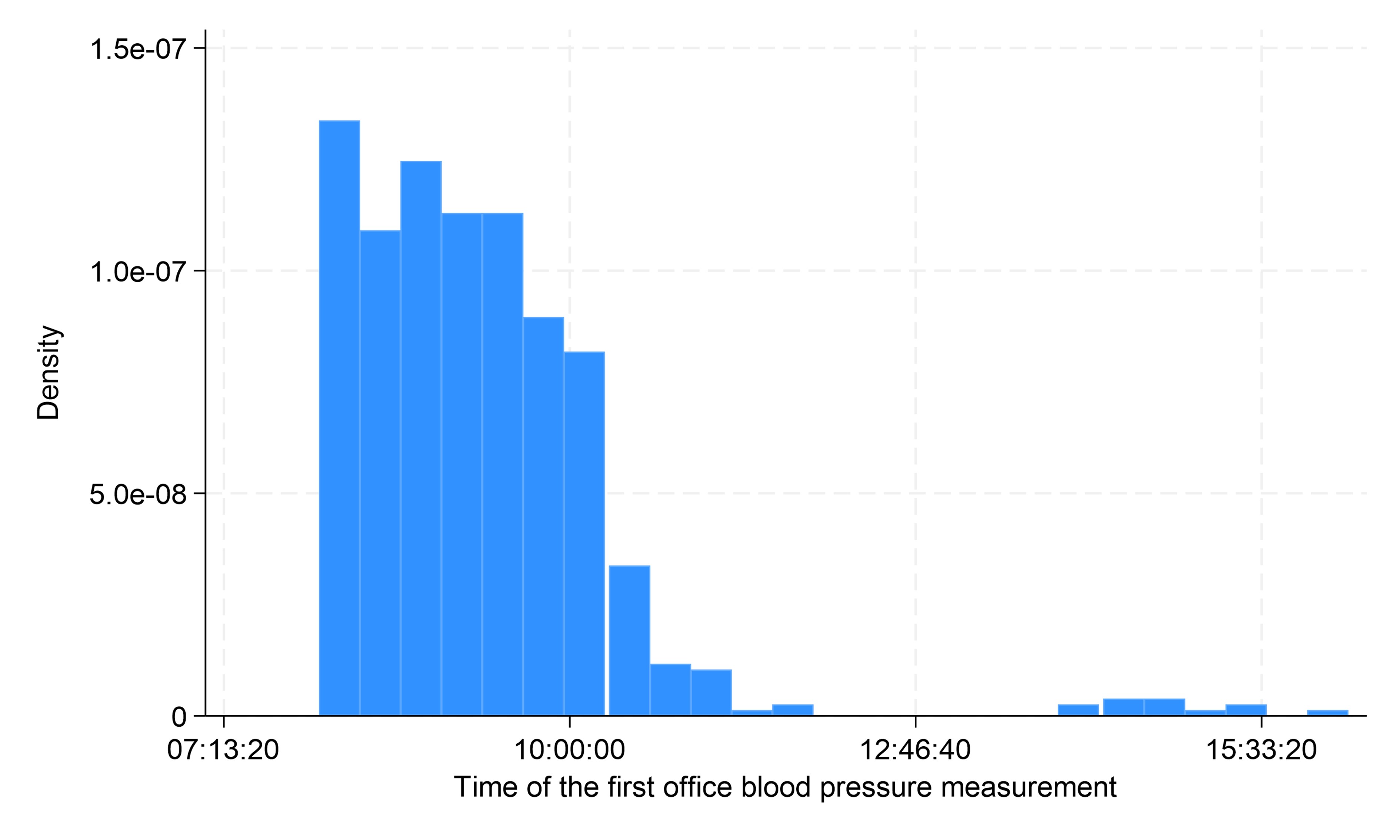

Supplement: Supplementary Figure 2 — Distribution of time of the first office blood pressure measurement. [file Image_2.jpeg]

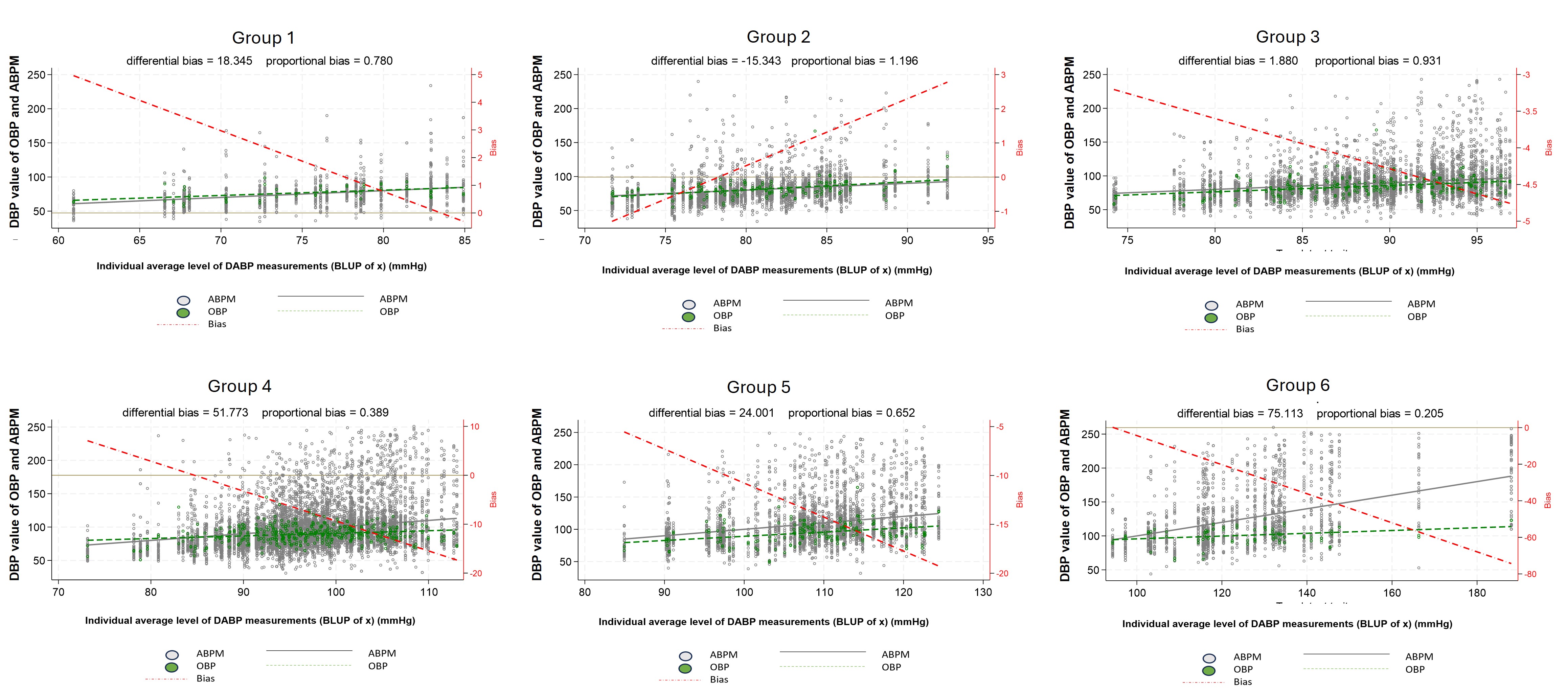

Supplement: Supplementary Figure 3 — Difference between diastolic office blood pressure and diastolic daytime ambulatory blood pressure by groups of BP level. Group 1: 100 ≤ true average SBP < 120 mmHg or 60 ≤ true average DBP < 80 mmHg, Group 2: 120 ≤ true average SBP < 130 mmHg or 80 ≤ true average DBP < 85 mmHg, Group 3: 130 ≤ true average SBP < 140 mmHg or 85 ≤ true average DBP < 90 mmHg, Group 4: 140 ≤ true average SBP < 160 mmHg or 90 ≤ true average DBP < 100 mmHg, Group 5: 160 ≤ true average SBP < 180 mmHg or 100 ≤ true average DBP < 110 mmHg, Group 6: true average SBP ≥ 180 mmHg or true average DBP ≥ 110 mmHg, White dot: individual blood pressure per subject (ABP), Green dot: individual blood pressure per subject (OBP), Dash red line: regression line of average difference between OBP and ABPM values, Plain black line: mean ABPM value (all patients), Dash green line: mean OBP value (all patients), ABPM, daytime ambulatory blood pressure; OBP, office blood pressure; DBP, diastolic blood pressure. For diastolic blood pressure only blood pressure value ranging from 40 to 150 mmHg were considered. [file Image_3.jpeg]

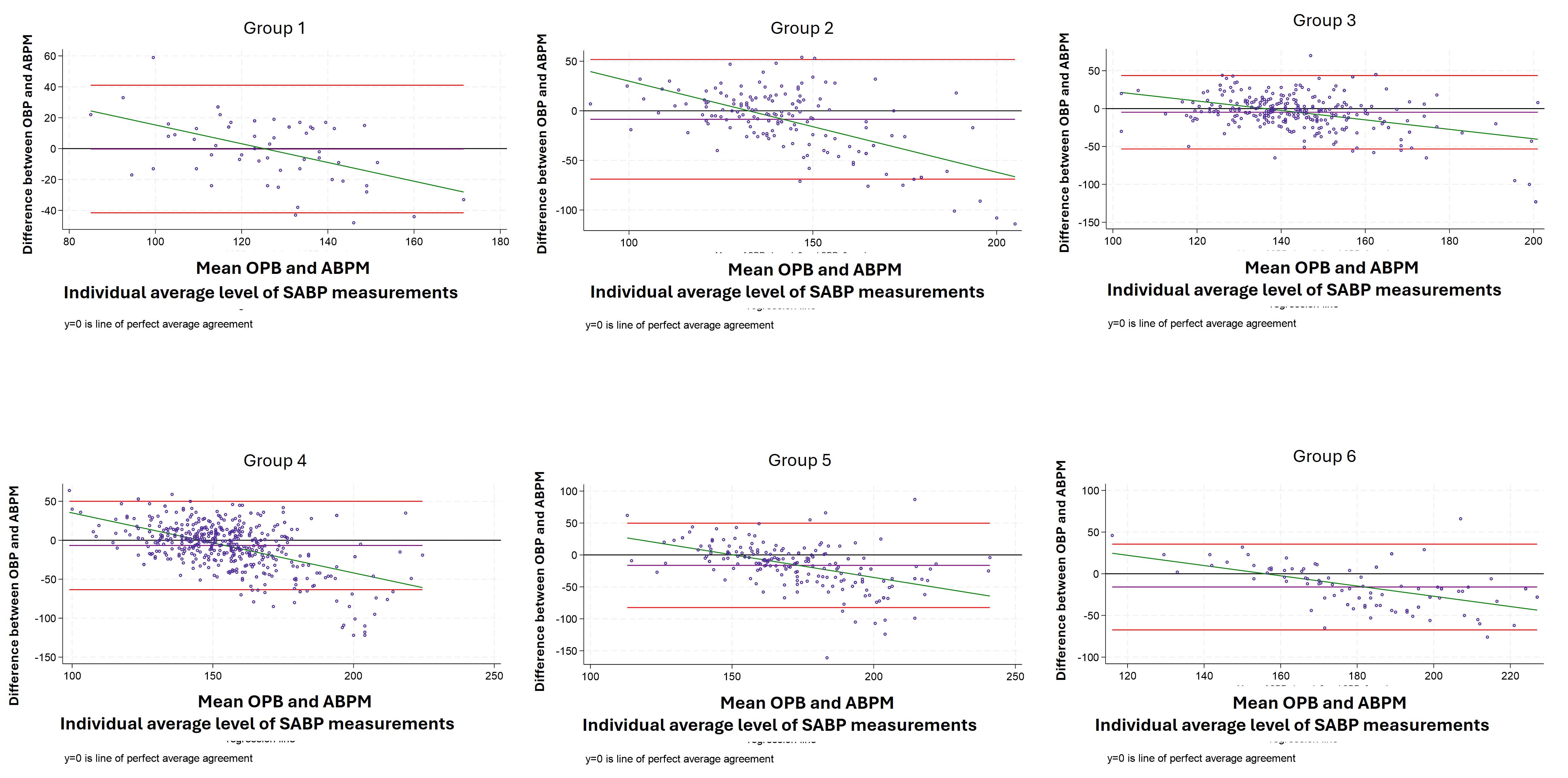

Supplement: Supplementary Figure 4 — Difference between systolic office blood pressure and systolic daytime ambulatory blood pressure by groups of BP level using the Bland and Altman methodology. Group 1: 100 ≤ true average SBP < 120 mmHg or 60 ≤ true average DBP < 80 mmHg, Group 2: 120 ≤ true average SBP < 130 mmHg or 80 ≤ true average DBP < 85 mmHg, Group 3: 130 ≤ true average SBP < 140 mmHg or 85 ≤ true average DBP < 90 mmHg, Group 4: 140 ≤ true average SBP < 160 mmHg or 90 ≤ true average DBP < 100 mmHg, Group 5: 160 ≤ true average SBP < 180 mmHg or 100 ≤ true average DBP < 110 mmHg, Group 6: true average SBP ≥ 180 mmHg or true average DBP ≥ 110 mmHg, Violet line: mean difference between OBP and ABPM, Red lines: 95% limits of agreements, Green line: regression line. OBP, office blood pressure; ABPM, daytime ambulatory blood pressure. [file Image_4.jpeg]
